# Cross-Scene Weakly Supervised Aircraft-Door Detection and Contour Extraction with Limited Pixel-Level Annotations

**DOI:** 10.3390/jimaging12100506

**Published:** 2026-10-13

**Authors:** Lexian Liang, Hailong Pei

**Affiliations:** 1 Key Laboratory of Autonomous Systems and Network Control, Ministry of Education, Guangdong Engineering Technology Research Center of Unmanned Aerial Vehicle Systems, South China University of Technology, Guangzhou 510641, China; 18676003383@163.com; 2 Guangzhou Genye Information Technology Co., Ltd., Guangzhou 511436, China

**Keywords:** aircraft-door detection, contour extraction, passenger boarding bridge, weak supervision, limited annotation, cross-scene generalization, boundary evaluation

## Abstract

Reliable aircraft-door perception is essential for automatic passenger-boarding-bridge (PBB) docking, where small sill-localization errors can affect alignment safety. We formulate aircraft-door perception as cross-scene joint detection and contour extraction using abundant boxes and limited contour annotations. Unlike conventional region segmentation, the target is a thin, often open door-frame contour. CS-DoorContour combines box-supervised detection, soft-contour supervision, selectively valid region assistance, box projection, and uncertainty-aware cross-scene consistency. A versioned label pipeline converts antialiased annotations into one-pixel centerlines, soft distance targets, fixed-width bands, and region masks only when safe closure is possible. Capture-sequence-disjoint protocols evaluate in-domain, cross-camera, day-to-night, and leave-one-aircraft-instance-out generalization using boundary, distance, and docking-oriented geometry metrics. Across three seeds with 20 contour labels, the in-domain model obtains 93.92±3.23 Box AP, 0.876±0.002 BF1@2, 0.233±0.006 Boundary AP, and 1.47±0.22-pixel mean sill error. Across held-out-camera folds, Box AP ranges from 91.09 to 95.87 and BF1@2 from 0.829 to 0.855; held-out-aircraft folds obtain 72.30–86.43 Box AP and 0.755–0.865 BF1@2. The method reduces dense annotation requirements while preserving contour and sill geometry for practical docking.

## 1. Introduction

Passenger boarding bridges (PBBs) form the enclosed connection between an airport terminal and an aircraft cabin. Docking is conventionally performed by an operator who must align the bridge head with the passenger door while maintaining clearance from the fuselage, engines, and other ground equipment. Automating this operation can reduce repetitive workload and turnaround variability, but it also transfers a safety-critical part of the approach procedure to the perception system. Existing automatic-docking prototypes therefore combine cameras or range sensors with a controller that repeatedly estimates the target door position during approach [1]. Reliable visual acquisition is necessary throughout this process: loss of the door, a displaced target, or an incorrect estimate near the fuselage can interrupt docking or create a collision risk.

Aircraft-door perception has commonly been posed as object detection. This formulation is appropriate during the initial approach because a bounding box supplies a stable target center and scale at a relatively low annotation cost. Recent YOLO-based studies have demonstrated that learned detectors can locate cabin doors under realistic airport conditions [1,2]. A box, however, describes only the spatial extent of the door. It does not identify the lower sill, distinguish the exterior frame from an internal handle, or recover the local inclination and lateral boundary geometry. These quantities become more relevant as the bridge nears the aircraft, when the useful output is no longer merely “where is the door?” but “where is the physical boundary to which the bridge must align?” A filled region mask contains more spatial information than a box, yet its area-oriented representation is also indirect for this purpose. We therefore treat the visible door-frame contour, particularly its side edges and lower sill, as the primary fine-grained target.

**Figure 1:**
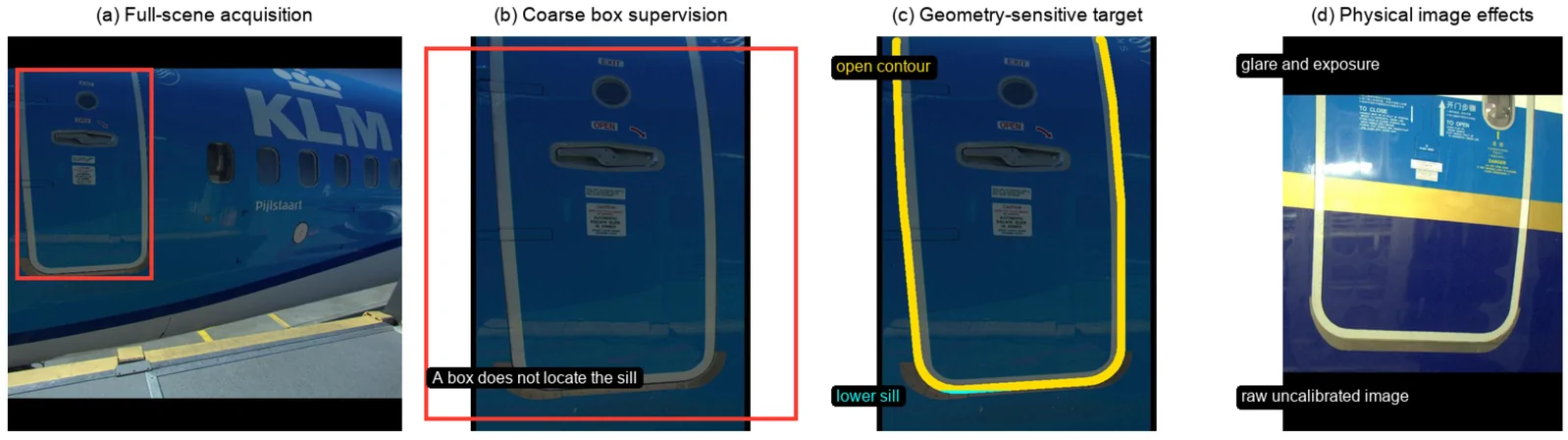
Aircraft-door perception for PBB docking. (**a**) Full-scene detection provides target acquisition. (**b**) A crop box does not determine the lower sill or distinguish exterior and internal structures. (**c**) The target annotation is a thin, potentially open exterior contour; yellow denotes the frame and cyan the lower sill. (**d**) Raw observations contain glare, exposure changes, and uncorrected lens geometry.

Figure 1 illustrates the resulting distinction between acquisition and geometric localization. The full-scene box identifies the target door, but the crop still contains internal handles, text, reflections, and other edges. The annotated target is a thin exterior frame that may remain open at the upper crop boundary, with the lower sill retained as a docking-oriented landmark. Raw nighttime observations additionally exhibit glare, exposure variation, and uncorrected camera geometry.

This target creates an annotation imbalance. Boxes can be produced rapidly and often already exist in detection datasets, whereas tracing a narrow door frame requires substantially more care. The problem is compounded by the semantics of the available labels: many aircraft-door traces are intentionally open at the top because the upper frame is outside the crop or visually ambiguous. Closing every trace to construct a conventional region mask would insert a boundary that was never observed. The learning problem is consequently neither ordinary instance segmentation nor pure edge detection. It must exploit abundant coarse boxes, learn an instance-associated contour from few precise traces, and use filled-region information only when closure is geometrically justified. Box-supervised instance segmentation shows that projection or affinity constraints can recover the extent of objects from inexpensive annotations [3,4], but reflective fuselage paint, decals, windows, and shadows weaken the appearance assumptions behind generic foreground grouping.

Generalization is an equally important constraint. A PBB-mounted camera observes changes in stand geometry, camera angle, aircraft type and livery, daylight, artificial lighting, reflection, exposure, blur, and partial occlusion. Domain-generalized segmentation research has shown that appearance-specific features can cause large performance losses on unseen visual domains and has addressed this through normalization, style diversification, or semantic invariance [5,6,7]. For aircraft doors, the desired contour geometry should remain stable under many of these appearance changes. Consistency learning offers a way to encode this prior while using images without contour labels [8,9,10]. Nevertheless, hard pseudo masks can amplify small systematic offsets along a thin boundary. A soft, uncertainty-aware target is better suited to pixels whose exact membership is ambiguous.

Dataset construction also affects the credibility of a cross-scene claim. Airport imagery is frequently extracted from video, so neighboring frames share nearly identical viewpoints, illumination, and background. Random image splitting can place adjacent frames in both training and test sets, allowing a model to appear robust while effectively seeing the same capture sequence during training. We address this issue by treating the capture sequence as the indivisible split unit and by separating in-domain, cross-camera, day-to-night, and cross-aircraft-instance protocols. This design evaluates distinct deployment shifts instead of reporting one aggregate out-of-domain score whose cause is difficult to interpret.

Modern transformer architectures provide a practical foundation for combining the coarse and fine tasks. DETR introduced set-based end-to-end detection [11]; DINO improved query initialization and denoising training [12]; and Mask2Former and Mask DINO unified high-quality mask prediction with transformer queries [13,14]. We build on this shared-query design but change the supervision hierarchy: detection provides acquisition, a selectively valid region provides global shape context, and the standardized contour remains the primary geometric output. Evaluation likewise emphasizes boundary-sensitive and docking-oriented quantities rather than relying on region IoU alone, which can obscure boundary errors [15].

Calibrated photogrammetric alternatives, including homography, PnP, stereo, and multi-view reconstruction, can recover metric door pose when camera intrinsics, distortion parameters, known door geometry, or cross-view correspondences are available [16]. These requirements are not met by the present raw monocular dataset. Our contribution is therefore complementary: it provides annotation-efficient, cross-scene two-dimensional door acquisition and open-contour landmarks that can serve as observations for a separately calibrated pose-estimation module, rather than claiming to replace photogrammetry or to estimate three-dimensional docking pose directly.

This work therefore studies cross-scene aircraft-door detection and contour extraction with abundant box annotations and limited contour annotations. The main contributions are as follows:

- We formulate a low-contour-annotation aircraft-door perception task for automatic PBB docking, using all available boxes while varying the number of contour labels.
- We define a reproducible contour-standardization pipeline that preserves open door-frame annotations and uses filled-region supervision only for contours that can be closed safely.
- We propose a unified detection–region–contour framework with box projection and uncertainty-aware cross-scene consistency, and characterize the detection–contour conflict caused by explicit geometry losses.
- We construct capture-sequence-disjoint in-domain and cross-scene protocols and an evaluation suite covering boundary accuracy, contour distance, door geometry, sill error, and deployment efficiency.

## 2. Related Work

### 2.1. Aircraft-Door Perception for PBB Docking

Automatic PBB docking requires the bridge head to acquire the door and then approach a docking pose without damaging the fuselage. Existing vision studies have primarily formulated the perception stage as aircraft-door object detection. Weon et al. used YOLO detections as target points for autonomous PBB motion and emphasized reducing input-image variation across environments and times [1]. Liu et al. introduced attention into a lightweight YOLOv5 detector to improve aircraft-door localization [2]. These studies establish the value of learned door acquisition, but a rectangular detection does not identify the lower sill or the local door-frame orientation. Our task therefore extends aircraft-door detection to a second, geometry-sensitive contour stage and evaluates errors that more directly describe final alignment.

Projective geometry provides the appropriate framework for recovering metric camera or object pose from calibrated views, correspondences, and known scene structure [16]. Such photogrammetric estimation is complementary to, rather than replaced by, the present task. The available dataset contains monocular image sequences but no camera intrinsics, distortion coefficients, multi-view correspondences, or metric three-dimensional door-pose ground truth. We therefore restrict the claimed output to two-dimensional door acquisition and image-space contour geometry. In a complete docking system, these predictions can provide door-frame landmarks or correspondences to a separately calibrated homography, perspective-*n*-point, stereo, or multi-view pose-estimation module.

### 2.2. Joint Object Detection and Segmentation

Two-stage instance segmentation attaches a mask predictor to detected regions, as exemplified by Mask R-CNN [17], while encoder–decoder models such as DeepLabv3+ recover dense boundaries using multiscale context [18]. DETR introduced set prediction with bipartite matching for end-to-end detection [11], and DINO subsequently improved convergence and accuracy through contrastive denoising, mixed query selection, and refined anchor prediction [12]. Mask2Former used masked attention to unify semantic, instance, and panoptic segmentation [13]; Mask DINO combined DINO queries with high-resolution pixel embeddings [14]. Our work adopts this shared-query paradigm but changes the primary pixel target from a filled object region to a standardized aircraft-door contour.

### 2.3. Learning Segmentation from Bounding Boxes

Box-supervised segmentation reduces annotation cost by converting spatial extent into weak pixel-level constraints. Early methods iteratively generated region proposals or foreground seeds from boxes [19,20,21]. BoxInst later introduced projection and pairwise affinity losses without modifying the segmentation architecture [3], while DiscoBox combined box supervision with a structured teacher and cross-image correspondence [4]. Projection is relevant to aircraft doors because the predicted structure should span the localized door extent; however, color affinity can be unreliable on reflective fuselage surfaces and multicolored liveries. We therefore combine box-derived projection with limited direct contour supervision and selectively valid region supervision.

Promptable foundation models provide a second way to convert a box into a dense mask. SAM and SAM 2 accept spatial prompts and transfer to images outside their original training distribution [22,23]. Their default output is a filled object region, whereas our target is a thin, frequently open frame contour. We therefore evaluate box-prompted segmentation as an external practical baseline and convert its predicted region boundary using the same frozen contour evaluator used for all methods.

Zhang et al. recently combined box-prompted SAM masks, photometric ensembling, uncertainty-aware pseudo labels, dynamic self-correction, and inference-time fusion for concrete-spalling segmentation [24]. That framework is well matched to compact filled defects, whereas an aircraft-door label in our task is a one-pixel exterior trace that may be intentionally open. We therefore use oracle-box SAM to test the direct promptable alternative and UniMatch to test generic semi-supervised learning, while preserving the target topology and evaluator rather than converting the annotation into a filled defect region.

Contour-coordinate methods such as Deep Snake and E2EC deform ordered closed polygons [25,26]. Their circular representation is not directly compatible with a trace that may terminate at the crop boundary: closing it inserts an unobserved top edge, while open-curve adaptation changes the representation, matching, and losses. We therefore do not report an unmodified polygon method. SAM ViT-B is the reproducible still-image promptable baseline. SAM 2 is discussed but not separately tested because independent crops provide no video propagation or temporal prompts; we do not assume equal numerical performance.

### 2.4. Boundary Learning and Cross-Scene Robustness

Classical deep boundary detectors such as HED, RCF, and BDCN learn multiscale side outputs or cascaded features to sharpen generic object boundaries [27,28,29]. Gated-SCNN separates shape and region streams, while SegFix refines labels near predicted boundaries [30,31]. PointRend treats boundaries as an adaptive point-sampling problem [32], and boundary-aware objectives address the severe class imbalance of thin structures [33]. Aircraft-door contours remain distinct because they are instance-associated and often intentionally open. Since localization errors dominate over area overlap, we use Boundary IoU/AP [15], surface distances, robust Hausdorff distance, and docking-specific geometry.

Consistency regularization supplies targets on sparsely labeled data by encouraging stable predictions under perturbations. Mean Teacher introduced a slowly varying soft target [8], and FixMatch combined weak-to-strong consistency with confidence filtering [34]. Segmentation-specific methods use perturbed decoders, mutual supervision, or uncertainty-aware pseudo labels [9,10,35,36,37]. Domain-generalization methods complement this strategy by varying feature statistics or enforcing semantic invariance [5,6,7]. In our setting, illumination, blur, local shadow, and partial occlusion should not change door geometry. We therefore use a detached soft prediction, weight pixels by teacher certainty, and map affine predictions into a common coordinate system when geometric perturbations are evaluated.

Recent difficulty-aware semi-supervised methods further refine how unreliable unlabeled samples are used. DSASS-Net combines difficulty-aware knowledge distillation, adaptive consistency, and stability-based sample selection for medical image segmentation [38], while DPLC-Net corrects difficult pseudo labels and adapts consistency weights and class-dependent thresholds for fine-grained medical image classification [39]. Although these methods address different imaging tasks, they reinforce the general principle that pseudo supervision should depend on sample reliability. Our method applies this principle at the pixel level by weighting aircraft-door contour consistency with teacher certainty.

Temporal evidence provides another route to robustness when video is available. HiGraph+ learns self-supervised space–time correspondences through graph-level and node-level structural consistency [40]; coarse-to-fine video instance segmentation uses factorized conditional appearance flows for association and refinement [41]; and SPOT stabilizes MLLM-generated segmentation prompts through explicit motion and geometric constraints [42]. These approaches motivate sequence-aware extensions of aircraft-door contour extraction. The present protocol nevertheless treats capture sequences as indivisible split units and performs inference on individual images, so we cite these methods as complementary directions rather than directly comparable baselines.

## 3. Problem Formulation

Let $D_{b}=\left\{\left(x_{i},b_{i}\right)\right\}$ denote the set of RGB images with door boxes and $D_{c}=\left\{\left(x_{j},b_{j},c_{j}\right)\right\}\subseteq D_{b}$ the smaller subset with contour annotations. A contour annotation $c_{j}\in {\left[0,1\right]}^{H\times W}$ is an antialiased door-frame trace and may be open where the top edge leaves the image. For a contour budget *K*, only a stratified subset $D_{c}^{K}$ is exposed to the contour loss, whereas all boxes remain available.

The model predicts a box $\hat{b}$, a contour probability map $\hat{c}$, and an auxiliary region map $\hat{r}$. The desired system must localize the door in known scenes, preserve accuracy under unseen cameras, lighting, and aircraft instances, and recover docking-relevant quantities such as the door center, lateral boundaries, inclination, and lower sill.

## 4. Materials and Methods

This section first describes the heterogeneous full-scene and door-crop data sources, then defines contour-label standardization and the sequence-disjoint evaluation protocols. It subsequently presents the shared detection–contour architecture, its partially supervised objectives, the limited-annotation protocol, and the evaluation metrics. This ordering separates data construction and model selection from the test results reported in Section 5.

### 4.1. Dataset and Annotation Types

The source directories contain 1957 detection-training images and 204 detection-validation images with YOLO-format boxes, together with 1693 contour-training and 210 contour-validation label files. These directory labels describe the original collection split rather than the sequence-disjoint experimental split introduced below. The contour PNGs are grayscale, antialiased door-frame traces rather than filled door interiors. Across the detection and contour sources, 2303 unique image names are available after duplicate names are merged.

Filename metadata encodes aircraft instance, acquisition date and time, camera or view identifier, and frame number for most samples. Three aircraft-instance identifiers (13,572, 15,071, and 16,091) occur in 1063, 489, and 709 unique images, respectively; 42 special prepared or measuring-door images have no reliable aircraft identifier.

### 4.2. Contour Label Standardization

All contour labels are processed using the frozen contour_standard_v1 specification. The grayscale label is thresholded at 192, and the largest eight-connected component is retained. Morphological skeletonization produces a one-pixel centerline *C*. A soft contour target is defined from the Euclidean distance transform d(p,C) as

(1) $$C_{\mathrm{soft}}\left(p\right)=\exp\left(-\frac{d{\left(p,C\right)}^{2}}{2\sigma^{2}}\right),\;\sigma =2.0.$$

Here, *p* is a pixel location, $d\left(p,C\right)=\min_{c\in C}{∥p-c∥}_{2}$ is its Euclidean distance to the one-pixel centerline, and σ is the Gaussian spread measured in pixels. We set σ=2.0 to match the radius-two (five-pixel-wide) hard contour band used by the fixed label-standardization pipeline. This preprocessing value was fixed before model training and was not selected using validation or test performance.

A five-pixel hard contour band is created by dilating the centerline with radius two. Region supervision is not assumed to exist for every image. A filled mask is valid only when the source contour is naturally closed or when exactly two endpoints lie within ten pixels of the upper image border and can be joined along that border. This yields 372 naturally closed and 274 safely top-border-closed masks; the remaining 1257 labels retain contour supervision but set the region-valid flag to false.

### 4.3. Sequence-Disjoint Cross-Scene Protocols

An audit of 171 capture sequences found that the original random split placed 118 sequences across training and validation, affecting 1462 unique images. One contour image name also appeared directly in both subsets. Consequently, the old split is used only for pipeline diagnosis.

The frozen versioned scene-split protocol assigns complete sequences rather than individual frames. The in-domain split contains 1868 training, 275 validation, and 160 test images. The day-to-night protocol uses 1267 daytime training images, 223 daytime validation images, and 771 unique night test images. Of these 771 images, 741 have full-scene detection annotations, and 625 have door-crop contour annotations; the two modality-specific subsets overlap and therefore do not sum to the number of unique images. Cross-camera evaluation uses three leave-one-camera-out folds with 1059, 233, and 415 unique test images for cameras 1–3. Cross-aircraft-instance evaluation similarly holds out identifiers 13,572, 15,071, and 16,091 in turn. The train, validation, and test sequence intersections are zero in every fold.

Data audit further showed that detection images are full-scene 640×640 frames, whereas contour images are door crops resized to 512×720. We therefore define a cascade: the detector localizes the door in the full frame, after which contour extraction operates in crop coordinates. Full-frame YOLO coordinates are never transferred directly to a contour crop.

The images are raw camera observations and were not corrected for lens distortion. Camera intrinsics and distortion coefficients are unavailable, so applying a physically valid undistortion transform or recovering metric three-dimensional pose is not possible for this dataset. The reported coordinates and errors are consequently image-space quantities. We treat distortion, glare, exposure, blur, and occlusion as deployment factors to be characterized rather than claiming that the input has been geometrically rectified.

### 4.4. Network Architecture

The proposed CS-DoorContour framework is based on a Mask-DINO-style shared transformer decoder with a ResNet-50 backbone. Features from res2–res5 feed a four-level pixel decoder; the three coarser levels enter a six-layer deformable encoder, and the shared query representation is refined by a six-layer transformer decoder. Detection queries predict class scores and door boxes, while the same representation interacts with a 256-dimensional, stride-4 pixel embedding to produce contour and region logits. Three output levels are used:

1. A detection head trained on every image with a box annotation;
2. A contour head trained on the limited standardized centerline and soft-contour targets;
3. An auxiliary region head trained only where the region-valid flag is true.

The region branch regularizes global door structure, whereas the contour branch remains the primary output at inference. The baseline uses 100 object queries and 20 denoising queries; subsequent modules retain these settings so improvements cannot be attributed to a larger decoder.

**Figure 2:**
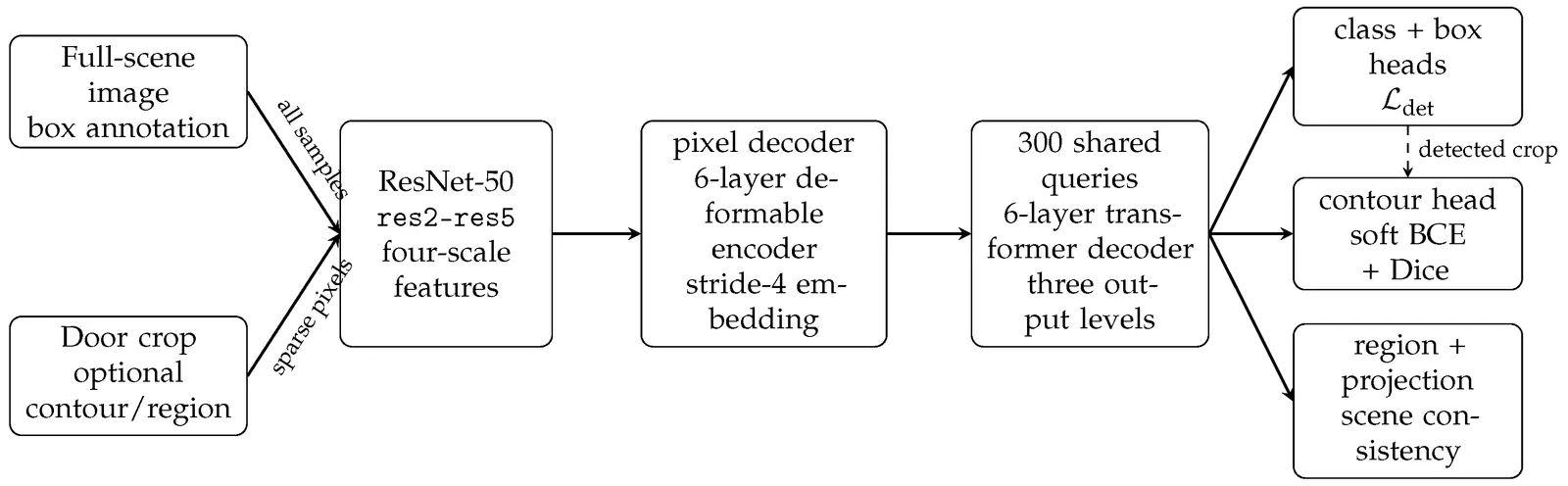
Architecture and supervision flow of CS-DoorContour. Unpaired full scenes and door crops share the backbone, multiscale pixel decoder, and transformer queries. Every sample contributes detection supervision; only genuine contour labels enter mask matching and contour losses, while valid closed regions, box projections, and coordinate-preserving teacher consistency provide auxiliary constraints. At inference, the full-scene detection defines the crop, and the crop contour is mapped back to image coordinates.

The two data domains are linked by model parameters rather than an assumed pixel correspondence. Full-scene images train acquisition and box localization, whereas standardized door crops train fine boundary geometry. At inference, the highest-scoring full-scene door box is expanded by a fixed context margin, rectified to the crop resolution, and passed to the contour head. Predicted contour coordinates are then transformed back to the full frame. This cascade permits abundant legacy boxes and scarce crop contours to be used jointly even when they were collected from different frames. The complete architecture and inference cascade are summarized in Figure 2.

### 4.5. Training Objectives

The total objective is

(2) $$L=L_{\det}+\lambda_{c}L_{\mathrm{contour}}+\lambda_{r}L_{\mathrm{region}}+\lambda_{p}L_{\mathrm{projection}}+\lambda_{g}L_{\mathrm{geometry}}+\lambda_{s}L_{\mathrm{scene}}.$$

$L_{\det}$ is the standard matched classification and box-regression objective. $L_{\mathrm{contour}}$ combines soft binary cross-entropy and Dice loss. $L_{\mathrm{region}}$ is masked out for invalid regions. Inspired by box-supervised segmentation [3], $L_{\mathrm{projection}}$ aligns horizontal and vertical projections of the predicted structure with the annotated box. Specifically, max projections of the sigmoid contour map along each image axis are compared by Dice loss with the corresponding one-dimensional box support. $L_{\mathrm{geometry}}$ constrains the center, robust extrema, aspect ratio, inclination, and sill. Finally, $L_{\mathrm{scene}}$ enforces soft agreement under photometric corruption, blur, noise, local shadow, overexposure, and partial occlusion.

In Equation (2), $\lambda_{c}=\lambda_{r}=1$; BCE and Dice inside each contour or valid-region loss are weighted 5 and 5. We use $\lambda_{p}=2$ and $\lambda_{g}=0$ (2 only in the shared-geometry diagnostic). The scene multiplier $\lambda_{s}$ is 0 for 262 updates, ramps linearly to 1 over the next 262, and remains 1; its mask, box, and class terms are weighted 1.0, 0.5, and 0.25. These archived settings were fixed across all scene folds without test-set tuning.

For scene consistency, the unperturbed prediction is detached and used as a soft teacher for a coordinate-preserving strongly perturbed view. The highest-confidence door query is selected independently in both views. For pixel *p*, teacher contour probability q(p), and student logit z(p), the contour term is

(3) $$\begin{array}{cc} L_{\mathrm{scene}}^{c}\; & =\frac{\sum_{p\in \Omega }w\left(p\right)\;\ell_{\mathrm{BCELogits}}\left(z\left(p\right),q\left(p\right)\right)}{\sum_{p\in \Omega }w\left(p\right)},\\ \ell_{\mathrm{BCELogits}}\left(z,q\right)\; & =-q\log s\left(z\right)-\left(1-q\right)\log\;\left(1-s(z)\right),\\ w(p) & =\max\;\left\{0.05,|2q(p)-1|\right\},\;s\left(z\right)=\frac{1}{1+e^{-z}}. \end{array}$$

Here, Ω is the crop-pixel lattice, s(·) is the sigmoid function, and $\ell_{\mathrm{BCELogits}}$ denotes binary cross-entropy evaluated directly from the student logit for numerical stability (the standard BCEWithLogits operation). The detached target satisfies q(p)∈[0,1], while z(p)∈R. The uncertainty weight w(p) approaches one for confident foreground or background targets and is lower-bounded by 0.05; hence the scalar denominator is strictly positive. The contour term is combined with $L_{1}$ box consistency and score consistency. Teacher queries below confidence 0.6 are ignored. Consistency starts after 262 warm-up updates and its weight increases linearly over the following 262 updates, reducing early confirmation bias.

For partial supervision, let *B* denote the minibatch size and let $m_{i}\in \left\{0,1\right\}$ indicate whether sample *i* has a genuine contour label. The direct baseline optimizes

(4) $$\begin{array}{cc} N_{c} & =\sum_{i=1}^{B}m_{i},\\ L_{\mathrm{direct}}\; & =\frac{1}{B}\sum_{i=1}^{B}L_{\det}^{\left(i\right)}+\lambda_{c}\frac{\sum_{i=1}^{B}m_{i}L_{\mathrm{contour}}^{\left(i\right)}}{\max\{1,N_{c}\}}. \end{array}$$

The quantity $N_{c}$ is the scalar number of genuinely contour-labeled samples in the minibatch. Thus, $\max\{1,N_{c}\}$ is the maximum of two scalars, not a maximization over a vector: it equals $N_{c}$ when at least one contour label is present and equals one otherwise. When $N_{c}=0$, every $m_{i}=0$, so the contour numerator and the entire contour term are zero.

The same indicator removes mask costs before bipartite matching, preventing a box-only compatibility mask from changing the query assignment. The normalization is synchronized across devices so that the contour-loss scale does not depend on how labeled crops happen to be distributed among GPUs.

Each training iteration is implemented in four steps. (1) A mixed minibatch is sampled from the abundant full-scene stream and the repeated sparse crop stream. (2) All samples pass through the shared backbone, pixel decoder, and transformer decoder, and classification and box losses are evaluated after bipartite matching. (3) For samples with $m_{i}=1$, genuine soft-contour and selectively valid region targets are added to matching and to the normalized pixel losses; compatibility rectangles from box-only samples remain excluded. (4) Projection is evaluated against box support, and a detached prediction of the weak view supervises the coordinate-preserving strong view after the warm-up and confidence test. Gradients from the active terms are accumulated once through the shared representation. This procedure does not require a full scene and contour crop to depict the same physical frame.

### 4.6. Limited-Contour Annotation Protocol

All training samples retain box supervision, while contour labels are restricted to budgets K∈{5,10,20,50,100,Full}. The frozen sequence-disjoint training pool contains 1517 eligible contour crops from 135 capture sequences. This number is smaller than the 1693 files in the original contour-training directory because the formal protocol reassigns complete sequences: 240 eligible crops are assigned to validation and 145 to testing across the complete 1902-image contour set, rather than preserving the original directory split. For each budget, samples are stratified by capture sequence, camera, lighting, aircraft instance, and contour closure status. The five budgets are nested within each of three seeds (20260729, 20260730, and 20260731), and every selected crop comes from a distinct sequence for K≤100. The sampled filenames are stored as versioned JSON manifests and shared by all competing methods.

### 4.7. Evaluation Metrics

Detection follows the COCO box AP protocol [43]. For an intersection-over-union threshold *t*, detections are ranked by confidence and matched one-to-one to ground-truth boxes when IoU≥t; ${\mathrm{AP}}_{t}$ is the area under the resulting interpolated precision–recall curve. Box AP averages ${\mathrm{AP}}_{t}$ over t∈{0.50,0.55,…,0.95}, while AP50 and AP75 expose coarse acquisition and stricter localization separately. Valid auxiliary regions are evaluated separately with region Mask AP. Box AP is reported only in the coordinate system in which its boxes are annotated and is not treated as a contour-quality measure.

Let *P* and *G* denote the predicted and ground-truth one-pixel contour sets, and let $d\left(p,G\right)=\min_{g\in G}{∥p-g∥}_{2}$. At pixel tolerance τ, boundary precision and recall are

(5) $$P_{\tau }=\frac{\left|\{p\in P:d(p,G)\leq \tau \}\right|}{\left|P\right|},\;R_{\tau }=\frac{\left|\{g\in G:d(g,P)\leq \tau \}\right|}{\left|G\right|},$$

and ${BF1}_{\tau }=2P_{\tau }R_{\tau }/\left(P_{\tau }+R_{\tau }\right)$. We report tolerances of 1, 2, 3, and 5 pixels following contour-matching practice [44]. Boundary IoU is computed after dilating both contour sets by the stated tolerance; Boundary AP then applies the same confidence-ranked precision–recall construction over Boundary-IoU thresholds 0.50:0.05:0.95 [15]. BF1 describes tolerance-based local correspondence, whereas Boundary AP penalizes duplicate or low-confidence instance predictions and evaluates performance across increasingly strict overlap thresholds.

Distance metrics retain geometric information that thresholded overlap discards. Average symmetric surface distance is

(6) $$\mathrm{ASSD}\left(P,G\right)=\frac{1}{2}\left(\frac{1}{\left|P\right|}\sum_{p\in P}d\left(p,G\right)+\frac{1}{\left|G\right|}\sum_{g\in G}d\left(g,P\right)\right).$$

HD95 is the 95th percentile of the pooled directed distances {d(p,G):p∈P}∪{d(g,P):g∈G}. It captures severe tail displacement while being less sensitive than maximum Hausdorff distance to a single isolated pixel [45].

Docking-oriented metrics include center error, left and right boundary errors, top and lower-sill errors, width and height errors, and inclination error. Spatial errors are reported both in pixels and normalized by door width, door height, door diagonal, or image diagonal as appropriate. Robust 1st and 99th percentiles define contour extrema. If S(P) denotes the points whose vertical coordinates fall in the lowest 2% of the predicted contour range, the predicted sill is ${\hat{y}}_{\mathrm{sill}}={\mathrm{median}}_{p\in S(P)}y_{p}$, and sill error is $|{\hat{y}}_{\mathrm{sill}}-y_{\mathrm{sill}}|$. Box AP therefore measures target acquisition, BF1 and Boundary AP measure thin-boundary recovery, ASSD and HD95 measure average and tail displacement, and sill error isolates the lower landmark most directly related to bridge alignment. Model parameters, single-image latency, frames per second, and peak inference memory are also reported.

## 5. Results

### 5.1. Implementation Details

Experiments use PyTorch 2.0.1, Detectron2 0.6, mixed-precision training, and four NVIDIA RTX 4090 GPUs. The DI-MaskDINO model is initialized from an ImageNet-pretrained ResNet-50. AdamW is used with base learning rate $10^{-4}$, weight decay 0.05, global batch size 16, full-model gradient clipping at 0.01, and a 0.1 backbone learning-rate multiplier. Door crops undergo large-scale jitter with scale range 0.5–1.5 and are padded or cropped to 512×512. Classification, mask BCE, Dice, box $L_{1}$, and GIoU weights are 4, 5, 5, 5, and 2, respectively. Full- and few-contour-only runs use 1138 updates; mixed-supervision runs use 1310 updates so that all abundant-box images are retained while the sparse contour stream is repeated. The corresponding evaluation intervals are 228 and 262 updates. For every learned contour configuration, the checkpoint with the highest validation contour AP is frozen before one-time testing; neither test contour metrics nor test detection AP participates in selection. Detection-only training uses the corresponding 12-pass schedule on the larger detection set. Unless a multi-seed aggregate is explicitly reported, the random seed is 20260729.

### 5.2. Baselines

The evaluated baselines comprise detection-only, a full-contour upper bound, few-contour-only learning, direct detection plus few-contour multi-task learning, region assistance, box projection, soft targets, and affine-mask consistency. External comparisons include oracle-box SAM and UniMatch. All learned internal comparisons use the frozen label version and sequence-disjoint splits; principal configurations are repeated across three seeds. The submitted single-seed teacher–student, shared-geometry, and affine box-consistency diagnostics are retained in the experiment archive but excluded from the repeated headline table.

The direct multi-task baseline pools the full-scene detection images and the selected contour crops without assuming pixel correspondence between the two domains. Every full-scene sample contributes classification and box-regression losses, whereas only a crop with a real contour annotation participates in mask-based Hungarian costs and contour losses. Compatibility rectangle masks used by the data loader are explicitly flagged and excluded from both operations. The two supervision streams are balanced by repeating the sparse contour subset while retaining every abundant-box sample; the total number of optimizer updates is held fixed. This baseline therefore tests whether shared representation learning from abundant boxes helps contour extraction before adding region, projection, geometry, or consistency constraints.

The repeated comparison establishes three main observations. First, abundant boxes remain useful beyond coarse acquisition: relative to the matched few-contour-only runs, direct multi-task learning increases BF1@2 from 0.837±0.013 to 0.844±0.016 and reduces sill error from 12.20±5.15 to 7.58±3.44 pixels. Second, selectively valid region and projection supervision further reduce sill error, but their detection AP varies across random seeds, so small single-run differences do not support a ranking claim. Third, soft targets stabilize the thin boundary. Appearance-only and affine-mask consistency are statistically similar: both attain BF1@2 of approximately 0.876 and sill error of approximately 1.5 pixels. We retain appearance-only consistency as CS-DoorContour because it avoids coordinate mapping and has lower Boundary-AP dispersion (0.006 versus 0.025 sample standard deviation), not because its mean is significantly higher. UniMatch collapses to all-background predictions, illustrating that generic semi-supervised region segmentation does not automatically address severe thin-contour imbalance; the oracle-box SAM result likewise shows that a generic closed region boundary is not equivalent to the target open frame.

Table 1 and Table 2 separate full-scene acquisition from crop-coordinate contour recovery; Section 5.6 isolates region, projection, soft targets, appearance consistency, and affine-mask consistency. Hard pseudo masks and explicit shared-feature geometry loss are excluded from the repeated headline comparison because their archived single-seed diagnostics respectively propagate boundary offsets and degrade detection. Numerical Box AP differences are interpreted only when the compared methods use the identical image set and coordinate system. Full-scene and crop-stage Box AP values are never compared directly.

### 5.3. Contour-Annotation Budget

**Figure 3:**
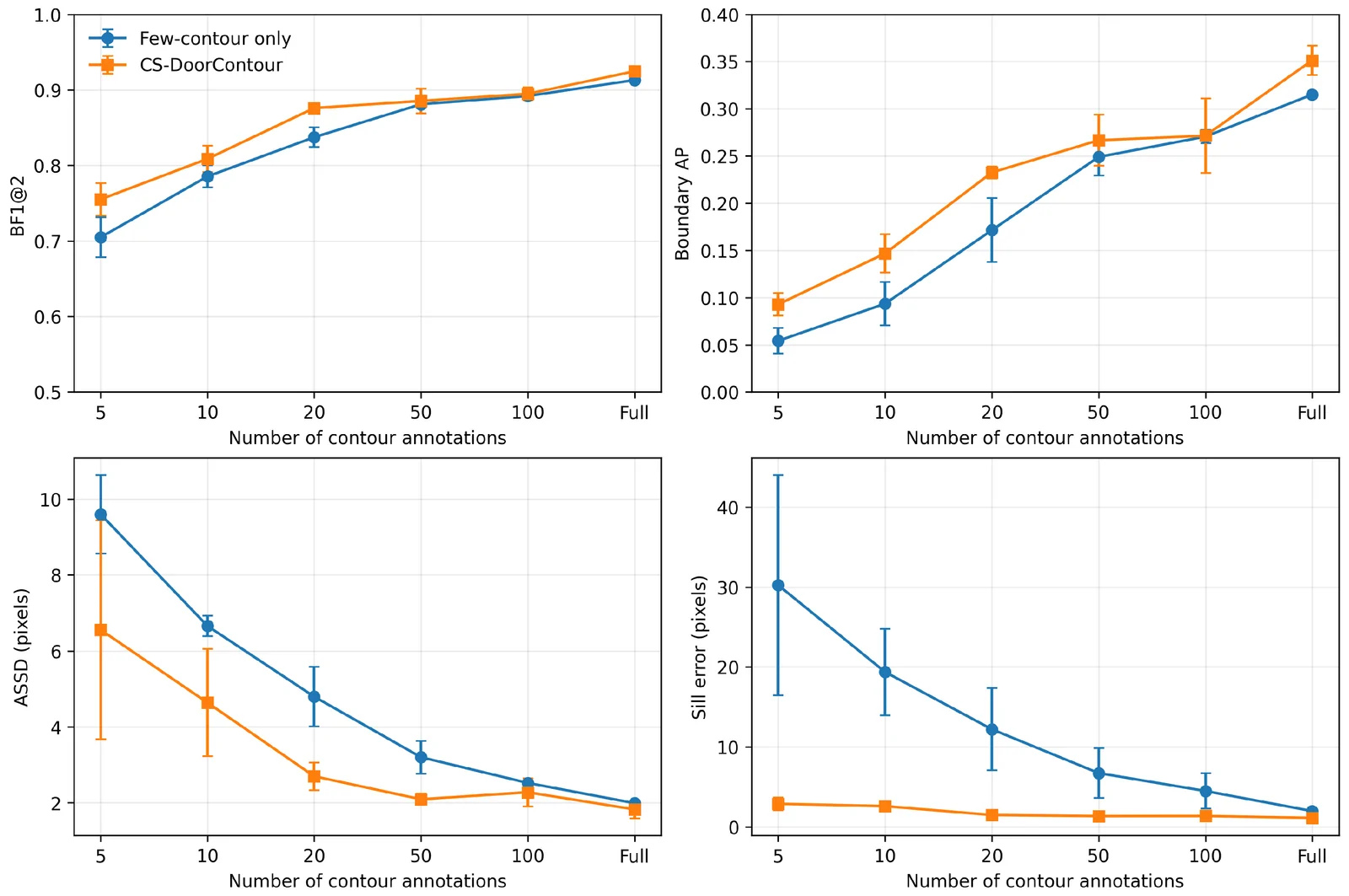
Annotation efficiency on the nested frozen subsets. Points and error bars show the mean and sample standard deviation across three seeds; the archived few-contour-only Full point is a single-run reference. Axis limits are fixed from the displayed quantities and do not use hidden data. CS-DoorContour is especially effective for lower-sill localization under small annotation budgets.

Table 3 summarizes all 18 proposed-method budget–seed experiments, each of which passed a completeness check requiring full-scene detection output and per-image contour metrics. From 5 to 50 annotations, mean BF1@2 rises from 0.755 to 0.885, while ASSD falls from 6.55 to 2.09 pixels. At 100 contours, mean BF1@2 reaches 0.895 and Boundary AP reaches 0.271 using 6.6% of the 1517 available contour labels. One 100-contour seed exhibits severe detection degradation (Box AP 19.33), producing the large 62.20±37.15 aggregate variation despite stable contour metrics. We retain this run rather than removing it post hoc; it shows that contour-checkpoint selection alone does not guarantee stable shared-query detection. Full contour supervision reaches 0.925±0.006 BF1@2 and 1.10±0.05-pixel sill error. Figure 3 compares the proposed method with the matched few-contour-only baseline and displays every evaluated point, including Full.

### 5.4. Cross-Scene Generalization

Table 4 reports every held-out domain separately so that scene-specific failure modes are not hidden by an aggregate mean.

The in-domain row establishes the frozen reference operating point for all cross-scene comparisons. The night test manifest contains 771 unique images, including 741 images with full-scene detection annotations and 625 door crops with contour annotations; these two evaluation subsets overlap. Day-to-night evaluation trains and selects the checkpoint using daytime data only, then tests the 741 full-scene detection images and 625 night contour crops. Box AP decreases by 3.45 points and BF1@2 by 0.030 relative to in-domain evaluation; ASSD and HD95 increase to 3.28 and 16.86 pixels. In contrast, the lower-sill error remains 1.62 pixels, indicating that the most docking-relevant vertical landmark is more stable under illumination shift than the complete contour and center geometry.

The three cross-camera folds obtain Box AP values of 91.09, 95.87, and 95.55 and BF1@2 values of 0.833, 0.855, and 0.829. Their small dispersion suggests consistent transfer across the viewpoints represented in this dataset. Leave-one-aircraft-instance-out transfer is harder: Box AP ranges from 72.30 to 86.43 and HD95 from 11.82 to 44.90 pixels. The 13,572 fold accounts for most of this variance, with 72.30 Box AP, 0.755 BF1@2, and 44.90-pixel HD95. Nevertheless, sill error remains between 1.32 and 2.58 pixels across the individual aircraft folds, substantially more stable than the complete-contour tail. These folds use the same no-target-test-access rule and are reported separately so that one aggregate score does not hide the visual factor that limits deployment.

### 5.5. Deployment Efficiency

Table 5 summarizes the measured latency, throughput, and device-memory requirements of the two deployment stages.

Both stages use the same shared model parameters and are executed sequentially in the deployment cascade. The measured rates exclude data loading and COCO metric accumulation, which are not part of online model computation. The full two-stage cascade requires approximately 108.6 ms per acquired door, corresponding to about 9.2 sequential inferences per second, while its maximum observed incremental device memory is 2.40 GiB. This measurement establishes computational feasibility on the tested GPU, not closed-loop control suitability: image acquisition, communication, tracking, pose estimation, and controller latency are outside present evaluation. A practical system can amortize the first stage by tracking the detected door between frames and invoking full-scene reacquisition only when confidence deteriorates.

### 5.6. Ablation Studies

Table 6 reports the controlled contribution of each auxiliary objective under the frozen 20-contour protocol.

No auxiliary objective improves every metric. Region supervision improves mean BF1@2 and reduces sill error, but its Box AP has high seed-to-seed variation. Projection further improves sill placement; adding the soft target reduces both mean sill error and its dispersion. Appearance consistency and affine-mask consistency produce nearly identical mean BF1@2 and sill error. Their mean Boundary AP values differ by only 0.003, whereas their sample standard deviations are 0.006 and 0.025, respectively. We therefore do not claim that appearance consistency is statistically superior. It is retained as the final configuration because it achieves the same contour–sill operating point without affine coordinate mapping. Single-seed geometry and affine box-consistency diagnostics from the submitted manuscript are excluded from this repeated headline table and treated only as exploratory evidence.

## 6. Discussion

Repeated experiments support the proposed supervision hierarchy while tempering the submitted single-run ranking. Adding abundant detection data to 20 contour labels reduces mean sill error from 12.20±5.15 to 7.58±3.44 pixels. Selectively valid regions regularize the global U-shape without fabricating an unobserved top edge, and projection further improves sill placement. The final appearance-consistent model reaches 1.47±0.22-pixel sill error and 0.876±0.002 BF1@2. Affine-mask consistency is statistically similar, so the evidence supports the supervision family rather than a strict ranking between these two consistency transformations.

The repeated annotation-budget study shows rapid early gains followed by diminishing returns. Increasing the budget from 5 to 50 contours reduces mean ASSD from 6.55 to 2.09 pixels; the 100-contour mean is slightly worse at 2.28 pixels and has overlapping variation. At 100 labels, BF1@2 reaches 96.8% of the fully supervised proposed-method result, while mean sill error is 1.34 pixels. The severe Box-AP failure in one 100-label seed also demonstrates that contour performance and full-scene acquisition can decouple under contour-only checkpoint selection. Thus, 20 contours provide a stringent low-label test, while 50–100 sequence-diverse annotations improve contour geometry but do not by themselves guarantee detection stability.

Cross-scene transfer and the controlled ablations expose complementary failure modes. Aircraft-instance transfer produces much larger HD95 variation than camera or illumination transfer, while sill error remains near two pixels. High box confidence does not guarantee a correct thin contour, and the SAM experiment shows that an oracle prompt can still produce the wrong closed topology. UniMatch instead converges to the dominant background class, exposing the class-imbalance difficulty of generic semi-supervised semantic segmentation for one-pixel targets. Soft appearance consistency and affine-mask consistency avoid these two failure modes and reach the same mean BF1@2; the coordinate-preserving appearance variant is used as the simpler final configuration.

**Figure 4:**
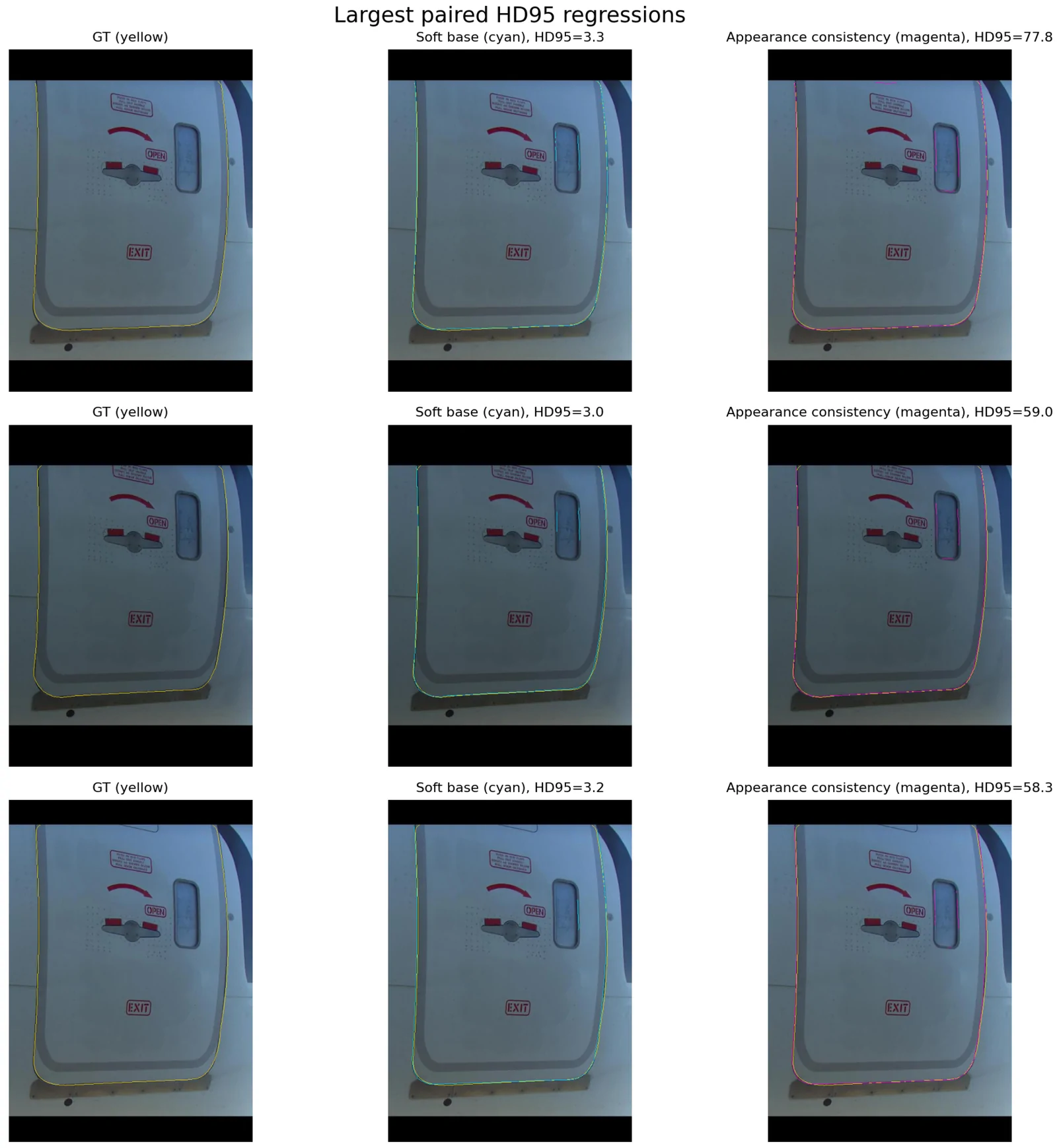
The three largest representative tail failures from one neighboring-frame sequence. Ground truth is yellow; the soft base prediction is cyan; and the appearance-consistent prediction is magenta. A disconnected response around the internal handle increases the 95th-percentile Hausdorff distance (HD95) despite close agreement along the exterior frame.

Tail analysis further explains why average overlap alone is insufficient. The increase in HD95 for appearance consistency is concentrated in six neighboring frames where an internal handle is predicted as a disconnected component. The median paired HD95 change is zero, and a box-interior component filter improves the aggregate diagnostic, but using that rule in the headline result would tune the test set. We therefore retain the unfiltered result and reserve component filtering for validation in the frozen cross-scene protocols. Figure 4 visualizes three representative frames from this failure sequence. This illustrates how sequence-aware error analysis can identify a repeated physical failure without overstating post hoc improvements.

Several limitations remain. The image-space contour does not resolve monocular scale ambiguity and has not yet been converted to calibrated 3D pose or closed-loop bridge-control error. Because the raw images were not undistorted and the camera intrinsics and distortion coefficients are unavailable, the effect of lens distortion on sill and side-boundary geometry cannot be removed retrospectively. A physically grounded pose comparison with photogrammetric methods would additionally require calibrated views, known door geometry or cross-view correspondences, and metric pose ground truth, none of which are available in the present dataset. The dataset contains only three identified aircraft instances, and some camera identifiers may mix semantically different viewpoints. Rare hazards, severe occlusion, precipitation, sun glare, and lens contamination are not represented sufficiently to support a safety claim. Finally, the present cascade is evaluated by stage; end-to-end propagation from an imperfect full-frame crop through calibrated pose estimation to final docking error requires a dedicated integration study. Consequently, CS-DoorContour should be viewed as a two-dimensional perception front end for a monitored docking system rather than a photogrammetric pose estimator or safety-certified controller.

## 7. Conclusions

This paper formulates aircraft-door perception for automatic PBB docking as joint detection and contour extraction under abundant box and limited contour supervision. The proposed pipeline preserves thin, often open contour semantics, avoids capture-sequence leakage, and evaluates both general boundary quality and docking-specific geometry. Across three seeds with only 20 contour annotations, CS-DoorContour obtains 93.92±3.23 Box AP, 0.876±0.002 BF1@2, 0.233±0.006 Boundary AP, 2.70±0.36-pixel ASSD, and 1.47±0.22-pixel sill error. Across individual held-out-camera folds, Box AP is 91.09–95.87 and BF1@2 is 0.829–0.855; held-out-aircraft evaluation is more challenging at 72.30–86.43 Box AP and 0.755–0.865 BF1@2. At 100 contours, mean BF1@2 reaches 96.8% of the fully supervised proposed-method result using 6.6% of the available contour labels, although one seed exhibits severe detection degradation. Single-stage inference runs at 17.2–19.8 FPS with at most 2.40 GiB incremental GPU memory. These findings favor a region-assisted, contour-primary formulation over either box-only detection or indiscriminate filled-mask supervision, while repeated runs caution against ranking closely matched consistency variants from one seed. Future work will connect image-space contour uncertainty to calibrated 3D door pose and closed-loop bridge control.

## Figures and Tables

**Table 1:** Full-scene aircraft-door detection on the identical 155-image sequence-disjoint test set. All rows use full-scene coordinates.

Method	Box AP
Detection only	93.72
Detection + few contours	88.60±5.39
Region-assisted contour	84.63±15.75
Region + box projection	92.18±5.64
Soft + region + projection	94.09±1.51
Affine-mask consistency	95.06±3.03
CS-DoorContour	93.92±3.23

Repeated configurations report mean ± sample standard deviation across seeds 20260729–20260731 on the identical 155-image full-scene test set. Detection only is the archived single-run acquisition reference and is not used for a repeated-run ranking. Crop-stage boxes are excluded.

**Table 2:** Door-contour extraction on the identical 145-image crop-coordinate test set.

Method	BF1@2	bAP	ASSD	Sill Err.
Full-contour upper bound ^†^	0.913	0.315	1.99	1.96
Few-contour only (20)	0.837±0.013	0.171±0.034	4.79±0.79	12.20±5.15
Detection + few contours	0.844±0.016	0.163±0.042	4.06±0.75	7.58±3.44
Region-assisted contour	0.866±0.008	0.214±0.024	2.97±0.40	3.74±1.43
Region + box projection	0.868±0.002	0.213±0.032	3.40±0.47	1.94±0.83
Soft + region + projection	0.871±0.003	0.222±0.015	2.86±0.55	1.57±0.05
Affine-mask consistency	0.876±0.002	0.236±0.025	2.69±0.39	1.48±0.26
CS-DoorContour	0.876±0.002	0.233±0.006	2.70±0.36	1.47±0.22
Box-prompted SAM ^‡^	0.602	0.00194	11.89	6.99
UniMatch ^§^	0.000	0.000	–	–

BF1@2, boundary F1 within 2 pixels; bAP, Boundary AP; ASSD, average symmetric surface distance; Sill Err., lower-sill error. Distance and sill errors are in crop pixels. Repeated learned configurations report mean ± sample standard deviation across three seeds on the identical 145-image test set. ^†^ Archived single-run fully supervised reference. ^‡^ SAM ViT-B receives the ground-truth crop box as an oracle prompt. ^§^ UniMatch converged to all-background predictions under the 20-label thin-contour setting; distance and sill metrics are undefined for an empty contour and are therefore not reported.

**Table 3:** Annotation-budget performance of CS-DoorContour. Values are mean ± sample standard deviation across three seeds.

Contours	Box AP	BF1@2	bAP	ASSD	Sill Err.
5	89.88±3.11	0.755±0.021	0.093±0.012	6.55±2.88	2.87±0.81
10	94.00±1.91	0.809±0.018	0.147±0.020	4.64±1.41	2.58±0.52
20	93.92±3.23	0.876±0.002	0.233±0.006	2.70±0.36	1.47±0.22
50	90.47±2.94	0.885±0.016	0.267±0.027	2.09±0.15	1.33±0.25
100	62.20±37.15	0.895±0.008	0.271±0.039	2.28±0.37	1.34±0.06
Full	91.09±3.02	0.925±0.006	0.351±0.015	1.82±0.23	1.10±0.05

**Table 4:** Single-fold cross-scene evaluation. Each row is one independently trained fold with a capture-sequence-disjoint test domain; no fold averages are mixed with single-run results.

Protocol	Box AP	BF1@2	bAP	ASSD	HD95	Sill Err.
In-domain	96.73	0.875	0.239	2.63	13.71	1.22
Day-to-night	93.28	0.845	0.185	3.28	16.86	1.62
Camera 1 held out	91.09	0.833	0.174	3.17	16.43	1.25
Camera 2 held out	95.87	0.855	0.196	2.10	9.08	1.26
Camera 3 held out	95.55	0.829	0.139	2.85	13.83	2.37
Aircraft 13,572 held out	72.30	0.755	0.096	6.73	44.90	2.58
Aircraft 15,071 held out	77.84	0.865	0.217	3.11	17.24	2.22
Aircraft 16,091 held out	86.43	0.865	0.214	2.53	11.82	1.32

All rows are single test results using seed 20260729. Box AP is evaluated on full-scene images; contour metrics are evaluated on rectified door crops and are not in the same image coordinates. Distance and sill errors are in crop pixels. The three camera folds and three identified-aircraft folds are too few to estimate population-level variability; their ranges describe only the held-out domains represented in this dataset.

**Table 5:** Single-image deployment efficiency on one NVIDIA RTX 4090. Latency is pure model computation after warm-up; peak memory is the incremental device memory measured during a complete frozen-set inference run.

Stage	Parameters (M)	Latency (ms)	Throughput (FPS)	Peak Memory (GiB)
Full-scene detection	55.99	50.4	19.8	2.40
Door-crop contour	55.99	58.2	17.2	2.26

**Table 6:** Repeated component ablation with 20 contour annotations. Values are mean ± sample standard deviation across three seeds. The ✓ indicates with the corresponding component.

Variant	Soft	Region	Proj.	Consistency	Box AP	BF1@2	Boundary AP	Sill Err.
Direct hard contour				None	88.60±5.39	0.844±0.016	0.163±0.042	7.58±3.44
Selective region		✓		None	84.63±15.75	0.866±0.008	0.214±0.024	3.74±1.43
Region + projection		✓	✓	None	92.18±5.64	0.868±0.002	0.213±0.032	1.94±0.83
Soft + region + projection	✓	✓	✓	None	94.09±1.51	0.871±0.003	0.222±0.015	1.57±0.05
CS-DoorContour	✓	✓	✓	Appearance	93.92±3.23	0.876±0.002	0.233±0.006	1.47±0.22
Affine-mask consistency	✓	✓	✓	Affine	95.06±3.03	0.876±0.002	0.236±0.025	1.48±0.26

## Data Availability

The original contributions presented in this study are included in the article and Supplementary Materials. Further inquiries can be directed to the corresponding author.

## Supplementary Materials

The following supporting information can be downloaded at: https://www.mdpi.com/article/10.3390/jimaging12100506/s1, Code S1: code.
